# *Jujubae Fructus* extract prolongs lifespan and improves stress tolerance in *Caenorhabditis elegans* dependent on DAF-16/SOD-3

**DOI:** 10.1038/s41598-024-64045-0

**Published:** 2024-06-14

**Authors:** Zhi Zhang, Jiajia Li, Feng Li, Tao Wang, Xiaoyan Luo, Bing Li, Yilin You, Changjing Wu, Xiaomeng Liu

**Affiliations:** 1 Department of Nutrition and Food Hygiene, College of Public Health, Xinxiang Medical University, 601 Jinsui Avenue, Xinxiang City, Henan Province China; 2 Key Laboratory of Animal Ecology and Conservation Biology, Institute of Zoology, Chinese Academy of Sciences, Beijing, China; 3 Institute of Translational Medicine, Zhoukou Normal University, No.6, Middle Wenchang Avenue, Chuanhui District, Zhoukou, China; 4 College of Food Science and Nutritional Engineering, Beijing Key Laboratory of Viticulture and Enology, China Agricultural University, Beijing, China

**Keywords:** *Jujubae Fructus*, *Caenorhabditis elegans*, Lifespan, Stress tolerance, *Daf-16*, Senescence, Molecular medicine

## Abstract

*Jujubae Fructus*, the fruit of *Ziziphus jujuba* Mill has been used as one of the medicine food homology species for thousands of years in China. Studies have shown that the active ingredients of *Jujubae Fructus* have a variety of biological effects, but its role in the aging process still lacks knowledge. Here, we investigated the effect of *Jujubae Fructus* extract (JE) on *Caenorhabditis elegans* lifespan and its potential mechanism. The lifespan of *C. elegans* treated with JE was signifificantly increased in a dose-dependent manner. In addition, JE treatment prolonged the reproductive period and increased normal activity during aging in *C. elegans*. Similarly, JE supplementation also enhanced the resistance to heat and oxidative stress in *C. elegans*. Furthermore, the mutant worms' lifespan assays demonstrated that JE requires *daf-16* to prolong lifespan. DAF-16::GFP analysis of TJ356 showed that JE treatment translocates DAF-16::GFP to nucleus in transgenic worms. By analyzing the downstream of *daf-16*, we identify that JE may regulate *sod3* downstream of *daf-16*. Mutant worms' lifespan and transgenic reporter gene expression assays revealed that increasing SOD-3 expression was critical for extending longevity in *C. elegans* with JE therapy. Collectively, these data indicate that JE may have an important role in *C. elegans* longevity that is dependent on DAF-16 and SOD-3.

## Introduction

Aging is the process of physiological and biochemical changes in the body and functional degradation with age^1^. As the major risk factor for most of chronic diseases, it increases the incidence and mortality of obesity, diabetes, cancer, neurodegenerative diseases, and cardiovascular diseases^2,3^. Therefore, delaying the aging process is of great significance to improve people's quality of life, preventing various diseases and even the sustainable development of society. Considering an explosive increase of senior citizens suffering from a number of chronic disorders, which could result in a tremendous social and economic burden, it is imperative to concentrate our collective efforts on developing novel remedies in order to decelerate senile changes and deter the inevitable physical alternations from advancing to the formidable ailments.

*Ziziphus Jujuba* is a plant belonging to Ziziphus species in the buckthorn family Rhamnaceae. Historically, it has been widely cultivated in Europe, southern/eastern Asia, and Australia for over 4000 years, mainly because the fruit of *Ziziphus Jujuba* (*Jujubae Fructus*) has many active ingredients. The main biologically active components of *Jujubae Fructus* include cAMP, phenolics, flavonoids, triterpenic acids, and polysaccharides^4^. In traditional Chinese medicine, *Jujubae Fructus* is often used to treat insomnia, spleen deficiency and palpitations, and used together with other medicinal materials to lower blood pressure, lower blood lipid and anti-anxiety. In the recent years, many studies have shown that the main biologically active components of *Jujubae Fructus* have a variety of biological effects^5–16^. Through comparative analysis of the hypnotic and sedative effects of flavonoids, saponins, and polysaccharides extracted from *Jujubae Fructus*, saponins have been proved to have superior hypnotic and sedative effects than flavonoids, and may exert excellent sedative-hypnotic effects simultaneously through a variety of mechanisms^5,6^. *Jujubae Fructus* can promote the learning and memory of the ovariectomized rats^7^, and repair the memory and behavior disorders caused by NBM lesion in rats^8^. It has been reported that *Jujubae Fructus* could protect the progression of CCl_4_-induced liver injury, which may be due to the fact that jujubae can bind to harmful free radicals and reduce their toxicity, as well as inhibit the inflammatory response of CCl_4_-induced liver injury^9,10^. The active components of *Jujubae Fructus*, such as triterpenic acid, also have anticancer activity and can induce cell death by inhibiting cell proliferation and apoptosis^11–13^. *Jujubae Fructus* also has an effect on glucose and lipid metabolism^14,15^. The *Jujubae Fructus* polysaccharides can improve insulin resistance and dyslipidemia induced by fructose in mice^14^. The infusion of *Jujubae Fructus* has beneficial effects on blood lipid and glycosylated hemoglobin in patients with type 2 diabetes mellitus^15^. There are few studies on the effect of *Jujubae Fructus* on aging, Ghimire et al. found that *Jujubae Fructus* feeding extended lifespan and health span of *drosophila*^16^.

About 60–80% of genes in *C. elegans* homologous to humans^17^, the generation cycle of *C. elegans* is three days, the average life span is about 2–3 weeks, and it is easy to culture and observe. These advantages make *C. elegans* an ideal model for aging research^17^. The insulin/insulin-like growth factor 1 (IIS) is the most classic signaling pathway that regulating the aging process of nematodes. *Daf-2* is one of the key genes, and inhibiting *daf-2* expression can significantly prolong the lifespan of nematodes. The key transcription factors that regulate the downstream lifespan of IIS are *daf-16*, *hsf-1*, and s*kn-1*, and their activation is beneficial for delaying aging. In the IIS signaling pathway, insulin or IGF-1 binds to DAF-2 receptor to induce the phosphorylation of PI3K/AGE-1, which activates the downstream AKT-1 pathway to phosphorylate DAF-16 and HSF-1, preventing transcription factor transfer into the nucleus, thereby shortening the nematode lifespan^18^. SKN-1 is Nuclear factor erythroid (NRF) transcription factor. When IIS protein kinase undergoes phosphorylation, SKN-1 remains in the cytoplasm and participates in the expression of detoxification and stress-related genes. SKN-1 can also promote protein homeostasis and prolong host lifespan by producing proteasomes. The heat shock response transcription factor HSF-1 can bind to specific regions of DNA containing heat shock factors^19,20^. The binding of HSF-1 to heat shock elements (HSEs) induces overexpression of genes encoding molecular chaperones HSP-70 and HSP-16, prolonging host lifespan, and enhancing stress response^21^. Numerous studies have focused on prolonging the lifespan of *C. elegans*, and the mechanism has been found to be related to *daf-16*. *Polygonum multiflorum* and *Agrimonia procera* extract have been shown to influence nematodes' lifespan via *daf-16*^22,23^. The longevity prolonging effect of blueberry on nematode and the enhancement of stress resistance were also confirmed to be mediated by *daf-16*^24^. The accumulation of ROS (reactive oxygen species) and oxidative stress injury in *Glochidion zeylanicum* treated nematodes were decreased, which was dependent on the DAF-16/FoxO and SKN-1/Nrf-2 signaling pathways^25^. In addition, *lyceum barbarum* polysaccharides, Liangyi Gao, *Holothuria leucospilota,* and tiger milk mushroom were also found to improve the health status and increase lifespan of *C. elegans* via the DAF-16 signaling pathway^26–29^. Therefore, *C. elegans* is not only an ideal model to study the aging phenotype, but is also widely used to study the anti-aging mechanism. As a traditional Chinese medicine, *Jujubae Fructus* can be taken as a food and medicine, and have a variety of biological effects^5–16^. There are limited studies on the effect of JE on lifespan, and the molecular mechanism of effects JE treatment based on *C. elegans* is still not clearly defined. Herein, to investigate the effect of JE treatment on *C. elegans*, we supplemented *C. elegans* with JE to investigate lifespan, vitality, fertility, and stress resistance of *C. elegans*, and further studied the mechanism of the effect of JE on *C. elegans*.

## Materials and methods

### Plant extract preparation

The dried ripe fruit of *Ziziphus jujube* (*Jujubae Fructus*) was obtained from Bozhou Chinese medicinal materials market and identified as *Zizyphus Jujuba* cv. Jinsixiaozao by Professor Hongmei Gu (Zhoukou Normal University). The voucher specimen (ZF-180621) was deposited in the Institute of Translational Medicine, College of Life Science and Agronomy, Zhoukou Normal University under closed and dry conditions at 25 ± 5 °C. *Jujubae Fructus* was cut up and extracted twice with boiling water for 2 h. The extract solution was filtrated immediately and the final filtrates were combined. The filtered extract was concentrated and dried under vacuum conditions by a rotary evaporator with 60 °C water bath. The total yield of water extract from *Jujubae Fructus* (JE) was 51.7% (w/w). The chemical stability of JE aqueous solution was evaluated with an accelerate test by heating up to 60 °C for 24 h, and comparative HPLC-PDAD-UV analysis was performed using an analytical Kromasil C18 column (5 μm, 100 Å, 4.6 mm × 250 mm; Akzo Nobel) on an Agilent 1100 HPLC system equipped with photo-diode array detector (G1316A). After filtered with 0.45 μm membrane, the JE sample solution in water (100 mg/mL) was injected (10 μL) into the column and eluted with a MeOH–water with 0.1% formic acid linear gradient (20% → 100% MeOH in 20 min followed by 5 min with isocratic 100% MeOH) mobile phase (flow rate 1.0 mL/min flow rate). The acquired photodiode array data (PDAD) data were processed with Agilent OpenLAB software.

### UHPLC-Q-TOF–MS/MS analysis of JE

JE sample (10 mg) was added 1 ml of 80% methanol. The mixture was ultrasonicated for 15 min (200 W, 40 KHZ) at room temperature, and then centrifuged for 10 min at 12,000 rpm. Finally, the supernatants were filtered through a 0.22 μm membrane to obtain prepared samples for UHPLC-Q-TOF–MS analysis. Chromatographic separation was performed in a Nexera UHPLC LC-30A system (Shimadzu Corporation, Jappan) equipped with a SHIMADZU InerSustain C18 (100 × 2.1 mm, 2 µm) column maintained at 35 °C with a flow rate of 1 mL/min. Gradient elution of analytes was carried out with acetonitrile (A) and 0.1% aqueous formic acid (B). The sample solution (5 μL) was injected after equilibration. An increasing linear gradient of solvent A (v/v) was then applied, as follows: 0–3 min, 5% A; 3–15 min, 5–20% A; 15–40 min, 20–100% A; 40–45 min, 100% A; 45–46 min, 100–5% A; 46–48 min, 5–5% A.

The ESI-MSn experiments were carried out on a TripleTOF 5600^+^ Hybrid Quadrupole-TOF LC/MS/MS Mass Spectrometer (AB SCIEX™, United States) with spray voltages of 5.5 and 4.4 kV in positive and negative modes, respectively. Ion source gas and curtain gas were set at 50 and 25 arbitrary units, respectively. The source temperatures were 500 and 450 °C in positive and negative modes, respectively. The analyzer scanned over mass ranges of m/z 100–1200 Da and m/z 50–1000 Da with accumulation time of 0.2 s and 0.01 s for TOF MS scan and product ion scan, respectively. Information-dependent acquisition (IDA) MS/MS experiments were performed with high sensitivity mode with Declustering Potential as ± 60 V and Collision Energy as 35 ± 15 eV. The analysis of UHPLC-MS data was performed using MS-DIAL 4.80 software (RIKEN Center for Sustainable Resource Science: Metabolome Informatics Research Team, Yokohama, Japan). We tentatively identified the compounds of JE by considering factors such as molecular weight, retention time, fragment information obtained from the MS/MS model, and further matching annotation in our prepared compounds, along with previous literature and the Traditional Chinese Medicine Systems Pharmacology (TCMSP) database.

### *C. elegans* strains and maintenance conditions

The *C. elegans* strains used were N2: Bristol (wildtype), CF1038: *daf-16* (mu86), CF1553: *sod-3* (muIs84), and DA1116: *eat-2* (ad1116), which were obtained from Shanghai Tech University. The PS3551: *hsf-1* (sy441), CB1370: *daf-2* (e1370), CL2070: *hsp-16.2* (dvIs70), EU31: *skn-1* (zu135), and TJ356: *daf-16* (zIs356) were obtained from Tongji University of Life Sciences and Technology. All the strains were maintained using standard conditions at 20 °C on NGM (nematode growth medium) plates. Worms were also allowed to grow in liquid S-medium with concentrated *Escherichia coli* OP50 (6 mg/mL) as a food resource. Eggs were extracted from the nematodes before all experiments to synchronize the nematodes (worms at spawning stage were collected in M9 buffer solution (containing 0.5 M NaOH and 0.8% NaClO), digested for 3–5 min and centrifuged to remove the supernatant at 1300×*g* for 30 s. The worms were washed twice with M9 buffer solution to retain the centrifuged precipitation^30^. When the worms reached L1 stage, Live or dead (heat inactivated) *E. coli* OP50 was added to the NGM plates as food to feed the worms. OP50 was killed by heat shock temperature control 75 °C during 2 h. In L4 stage, different concentrations of JE were added to the culture, and 40 μM Fluoro-2'-deoxy-β-uridine (FUDR, Sigma-Aldrich) was also necessary to inhibit the growth of progeny^30^.

### Toxicity assay

To perform the toxicity assay, the JE was dissolved in sterile water to prepare a stock solution of 100 mg/mL. The assay was performed using 100 μg/mL, 200 μg/mL, 1 mg/mL, 10 mg/mL, and 100 mg/mL concentrations. The control group was exposed to an equal volume of sterile water. On day 2 of adulthood, an age-synchronized population of worms was transferred to a 1 mL volume of S-medium supplemented with test doses of JE in a 12-well plate (Nest, China). A total of 90 worms were placed on triplicate wells with 30 worms per well for each group. The survival of worms was scored after every 2-h interval and the data shown represents the survival rate of worms after 24 h^31^.

### Lifespan assay

N2, CF1038, DA1116, PS3551, CB1370, EU31, and CL2070 lifespan assays were carried out at 20 °C. On day 2 of adulthood, an age-synchronized population of worms was transferred to NGM plates containing JE (20, 50, 100, 200 μg/mL) or an equal volume of sterile water (vehicle group). Each 3.5 cm plate was placed with 16–20 worms. Worms were transferred to new plates and scored every two days. Worms were scored as alive until there was no movement after repeated prodding. Lifespan assays were indicated in Supplementary Table S1, and the number of worms was pooled^32^.

### ROS assay

Wild-type N2 worms from the adult stage were treated with 100 μg/mL JE or vehicle and were used for intracellular ROS determination. At day 2 and day 4 adult worms age, synchronized worms were collected in 500 μL of 0.1% PBST buffer and then homogenized and sonicated. The sample using Fluorescent probe H2DCF-DA (2′,7′-dichlorodihydroflfluoresceindiacetate) and BioTek microplate reader at emission FL fluorescence intensity at 485 nm and excitation at 530 nm to measure emission FL fluorescence intensity. The observations were recorded for 90 min at intervals of 20 min. The assay was performed in triplicate independently.

### Lipofuscin assay

Wild-type N2 worms raised from the adult stage were treated with 100 μg/mL JE or vehicle used for lipofuscin assay. On the 10th day of adulthood (n = 20) were randomly selected and mounted on 3% agarose pads and anesthetized with 2% sodium azide. Images were captured with fluorescence microscope (Olympus BX 61, Japan) using GFP filter (with excitation at 340–380 nm and emission at 435–485 nm) FL fluorescence microscope at 10 ×. The fluorescence levels were quantified using the ImageJ software.

### Pharyngeal pumping rate

The pharyngeal pumping rate was quantified on the 5th, 7th, and 10th days of adulthood. Ten worms were treated with 100 μg/mL JE or vehicle randomly picked for measurement of pharyngeal pumping rate per 20  s.

### Movement assay

To measure the frequency of body bending, on day 2 of adulthood, an age-synchronized population of worms was treated with 100 μg/mL JE or vehicle for 2 and 11 days. Worms were placed on NGM plates containing FUDR (40 μM), and the number of sinusoidal curves made during locomotion and swinging head in 1 min was scored^33^.

### Fertility measurement

To determine fecundity, worms (N2/RNAi) were transferred to a medium without FUDR and treated with 100 μg/mL JE or vehicle treatment for 2 days. Hermaphrodites were serially transferred to fresh NGM plates at 24-h intervals, until sterile. Two days after eggs were laid, the number of hatched progenies was counted. When the nematodes were no longer postpartum, their reproductive cycles were recorded^34^.

### Heat stress resistance

For the heat stress resistance assay, on day 2 of adulthood, an age-synchronized population of worms was treated with 100 μg/mL JE or vehicle for 2 days. Live or dead OP50 (killed by heat shock temperature control 75 °C for 2 h) was added to NGM plates. Worms were placed on NGM plates containing FUDR (40 μM) in 37 °C conditions and then the number of worms was counted every hour until all the worms had died^35^.

### Oxidative stress resistance

On day 2 of adulthood, an age-synchronized population of worms was transferred to S-medium containing FUDR (40 μM) and with 100 μg/mL JE or vehicle treatment, respectively. After 2 days, 5 mM paraquat was added to S-medium, which induces lethal oxidative stress. The vitality of the worms was examined every 4 h until all worms had died. Triplicate plates were used for each group. Each group included 150 worms^24^.

### DAF-16 localization via fluorescence microscopy

The TJ356 strain was used to examine the localization of DAF-16 in the living nematode. The aged-synchronized L4 larvae were transferred to NGM plates previously treated with 100 μg/mL JE or vehicle and incubated for 48 h. DAF-16 localization was examined in 30 worms per treatment that were mounted in a drop of 20 mM levamisole hydrochloride. We scored each animal as having cytosolic localization, nuclear localization, or intermediate localization when there is a visible nuclear localization but one not as complete as nuclear. The number of worms with each level of nuclear translocation was counted. Fluorescence images were taken at constant exposure times (Olympus IX 73, Japan).

### Quantitative real-time PCR

The contemporaneous 2-day-old worms were incubated in NGM containing the same concentration of JE for 7 days. After washing with M9 buffer, *C. elegans* were collected into 1.5 mL tubes and extraction RNA using the TransZol Up (Transgen) and stored at − 80 °C. Complementary DNA was prepared using HiScript III 1st Strand cDNA Synthesis Kit (+ gDNA wiper) (Vazyme) for real-time polymerase chain reaction (RT-PCR). Quantitative PCR (qPCR) was performed using ChamQ Universal SYBR qPCR Master Mix (Vazyme). The mRNA expression levels of downstream genes of *daf-16* in nematodes were monitored. β-actin was used as the housekeeping gene for normalization, and the experimental results were expressed as 2−(ΔΔCt) values of qPCR. The information of the primer sequence is included in Supplementary Table S13.

### Statistical analysis

Statistical analyses were carried out using Graphpad 7.0. A Kaplan–Meier lifespan analysis was carried out, and p values were calculated using the log-rank test. Comparisons between groups were performed using Student’s t-test. In all statistical analyses, p < 0.05 was accepted as statistically significant (*p < 0.05, **p < 0.01, ***p < 0.001).

### Statement

The study complies with relevant institutional, national, and international guidelines and legislation.

## Results

### HPLC-PDAD-UV and UHPLC-Q-TOF–MS analysis of JE

**Figure 1:**
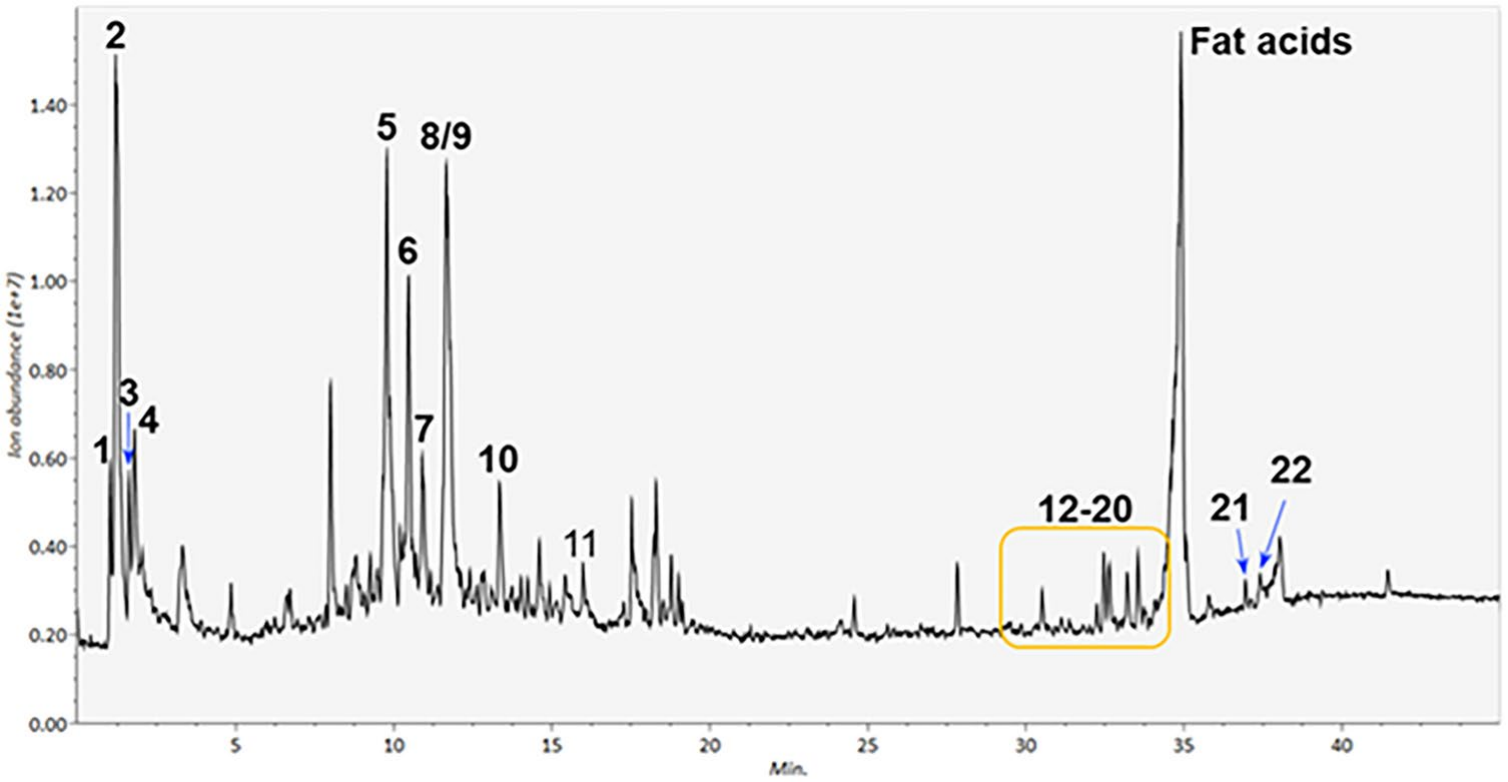
The chemical stability test for JE aqueous solution by HPLC-PDAD-UV analysis.

In the accelerated test, the fingerprint chromatograms derived from HPLC-PDAD-UV analysis of JE aqueous solution showed no significant changes after 24 h of heating (Fig. 1). Additionally, the UV absorption spectra of the main chromatographic peaks remained unchanged (data not shown). This result suggested that the JE is chemically stable in an aqueous solution during the lifespan assay under normal conditions. UHPLC-Q-TOF–MS analysis was conducted in both positive and negative modes, with Fig. 1 displaying the positive total ions chromatogram (TIC). A total of 22 phytochemicals were identified in JE, including three carbohydrates, five glycosides, two alkaloids, eleven triterpene acids, and cAMP (Table 1). Six of these compounds (cAMP, zizybeoside I and II, rutin, oleanonic acid, and ursolic acid) were identified by comparison to our previously prepared samples, the others were identified by comparison with literature^36,37^.

**Table 1:** Identified ingredients in JE.

No.	RT (min)	Compound name	Formular	MS	Error (ppm)	MS/MS
1	1.01	Tartaric acid	C_4_H_6_O_6_	151.0241 [M + H]^+^	1.32	110.0111, 99.0442, 88.2338, 82.0182
2	1.16	Methose	C_6_H_12_O_6_	179.0544 [M − H]^−^	6.70	125.1130, 101.0300, 89.0345
3	1.21	Sucrose	C_12_H_22_O_11_	341.1055 [M − H]^−^	8.50	163.0637, 145.0529, 127.0410
4	1.79	cAMP	C_10_H_12_N_5_O_6_P	330.0620 [M + H]^+^	-5.15	232.0850, 136.0638
5	9.77	Zizybeoside I	C_19_H_28_O_11_	431.1527 [M − H]^−^	6.03	347.0983, 293.1010
6	10.44	Zizybeoside II	C_25_H_38_O_16_	617.2054 [M + Na]^+^	0.65	431.1798, 269.1303, 161.0566
7	10.89	Coclaurine	C_17_H_19_NO_3_	286.1449 [M + H]^+^	− 2.10	269.1149, 237.0914, 209.0965, 175.0787, 145.0646, 107.0539
8	11.48	Zizyvoside II	C_31_H_50_O_18_	709.2858 [M − H]^−^	8.60	547, 519.2244, 385.2117
9	11.60	Stepharine	C_18_H_19_NO_3_	298.1451 [M + H]^+^	-2.68	269.1191, 192.1039, 161.0858
10	14.64	Rutin	C_27_H_30_O_16_	611.1597 [M + H]^+^	2.45	465.0975, 303.0503, 163.0670
11	16.11	Zizyvoside I	C_25_H_40_O_12_	533.2593 [M + H]^+^	0.94	435.1962, 393.1816, 277.2098
12	29.51	Ceanothic acid	C_30_H_46_O_5_	485.3259 [M − H]^−^	1.65	467.3810, 423.3476
13	30.51	Alphitolic acid	C_30_H_48_O_4_	473.3591 [M + H]^+^	8.45	455.3698, 390.9161
14	31.12	Maslinic acid	C_30_H_48_O_4_	495.3450 [M + Na]^+^	4.04	409.3433, 381.3086, 296.8615, 249.1672, 203.1779
15	31.38	2α-Hydroxyursolic acid	C_30_H_48_O_4_	473.3627 [M + H]^+^	0.85	437.3418, 409.3402, 391.3322, 285.2632, 223.1774, 205.1594, 187.1443
16	32.24	Zizyberanalic acid	C_30_H_46_O_4_	471.3475 [M + H]^+^	− 0.21	453.3368, 435.3225, 407.3355, 389.3332, 327.2283, 245.1501, 177.1640
17	33.20	3-O-cis-p-Coumaroylalphitolic acid	C_39_H_54_O_6_	619.3978 [M + H]^+^	3.39	437.3510, 391.3270, 259.1755, 202.5370, 173.1333, 135.1207
18	33.54	3-O-cis-p-Coumaroylmaslinic acid	C_39_H_54_O_6_	619.4019 [M + H]^+^	− 3.23	437.3447, 411.3292, 353.2572, 287.2157, 203.1819, 147.0457
19	34.07	3-O-trans-p-Coumaroylalphitolic acid	C_39_H_54_O_6_	619.4016 [M + H]^+^	− 2.74	437.3450, 409.3405, 391.3436, 363.2439, 201.1579, 177.1812
20	34.36	3-O-trans-p-Coumaroylmaslinic acid	C_39_H_54_O_6_	619.3944 [M + H]^+^	8.88	437.3506, 411.3207, 261.1825, 165.0583
21	36.94	Oleanonic acid	C_30_H_48_O_3_	455.3537 [M − H]^−^	− 2.64	437.3444, 409.3399, 259.1797, 177.1659
22	37.42	Ursonic acid	C_30_H_48_O_3_	455.3532 [M − H]^−^	− 1.54	437.3396, 409.3509, 261.1843, 208.1591, 163.1460

### Effect of JE on the lifespan and stress resistance of *C. elegans*

**Figure 2:**
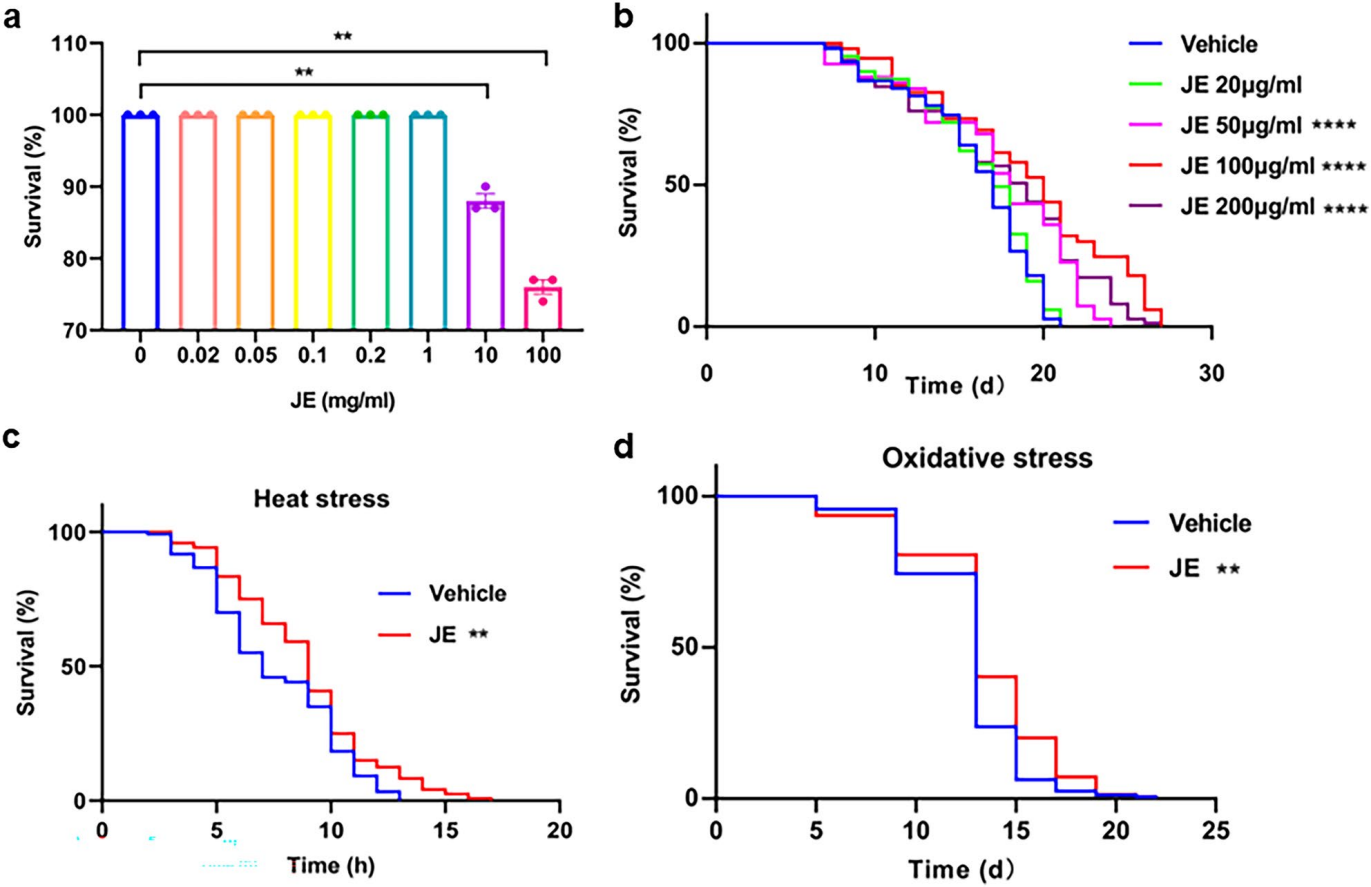
Lifespan and stress resistance of *C. elegans* exposed to JE. (**a**) Acute toxicity of JE on *C. elegans* feeding in S-medium. (n = 90). (**b**) Lifespan of *C. elegans* treated with 20 μg/mL, 50 μg/mL, 100 μg/ml and 200 µg/mL, or the corresponding concentration of vehicle. (n = 150). (**c**) Lifespan of *C. elegans* exposed to 37 °C thermal shock treated with 100 μg/mL JE or vehicle. (n = 120). (**d**) Lifespan of *C. elegans* exposed to 50 μM paraquat treated with 100 ug/mL JE or vehicle. (n ≥ 139). *P ≤ 0.05, **P ≤ 0.01, ***P ≤ 0.001 (vehicle vs treated). The experiment was carried out several times, and a typical trial is shown.

Before conducting lifespan experiments, we carried out an acute in vivo toxicity study. When the concentrations of JE were less than or equal to 1 mg/mL, which were found to be non-toxic to *C. elegans* were chosen for further tests (Fig. 2a). To address whether JE has a positive effect on the lifespan of *C. elegans*, N2 worms were treated with JE at treated with 20 µg/mL, 50 µg/mL, 100 µg/mL, and 200 µg/mL doses of standardized JE. The results showed that compared with the control group (equal volume sterile water was used as control), 50 µg/mL, 100 µg/mL, and 200 µg/mL JE treatment significantly increased the lifespan of the N2 worms in a dose-dependent manner, with the maximum lifespan increased from 21 days in control to 24, 27 and 27 days, respectively (Fig. 2b, Table S1). The mean lifespan significantly increased to 108.42%, 119.78%, and 110.18% with the treatment of 50, 100, and 200 μg/mL (Table S1). The 100 and 200 μg/mL doses of JE were able to extend the mean lifespan and maximum lifespan maximally. In the follow-up experiments, 100 μg/mL of JE was used to cultivate worms to observe its effects on other physiological indicators of *C. elegans*. Meanwhile, JE treatment also increased the lifespan of worms exposed to 20 °C or 37 °C thermal shock when fed bacteria killed by heat (Fig. S2). These data illustrated that JE-induced prolongation of lifespan occurs by a direct effect on *C. elegans* rather than indirectly through the bacteria.

Aging is often accompanied by a decline in resistance to stress, we exposed *C. elegans* to heat and oxidative stress to observe the effects of JE on *C. elegan's* lifespan. Under the thermotolerance conditions, the mean lifespan of the JE treatment group significantly increased by 16.37% compared with the control group. The maximum lifespan of the control group was 13 h, while that increased to 17 h after JE treatment (Fig. 2c, Table S2). The result of the oxidative stress showed JE treatment also could significantly increase the mean lifespan of *C. elegans* (Fig. 2d, Table S3).

### JE decreases the pigment lipofuscin and intracellular ROS level in *C. elegans*

**Figure 3:**
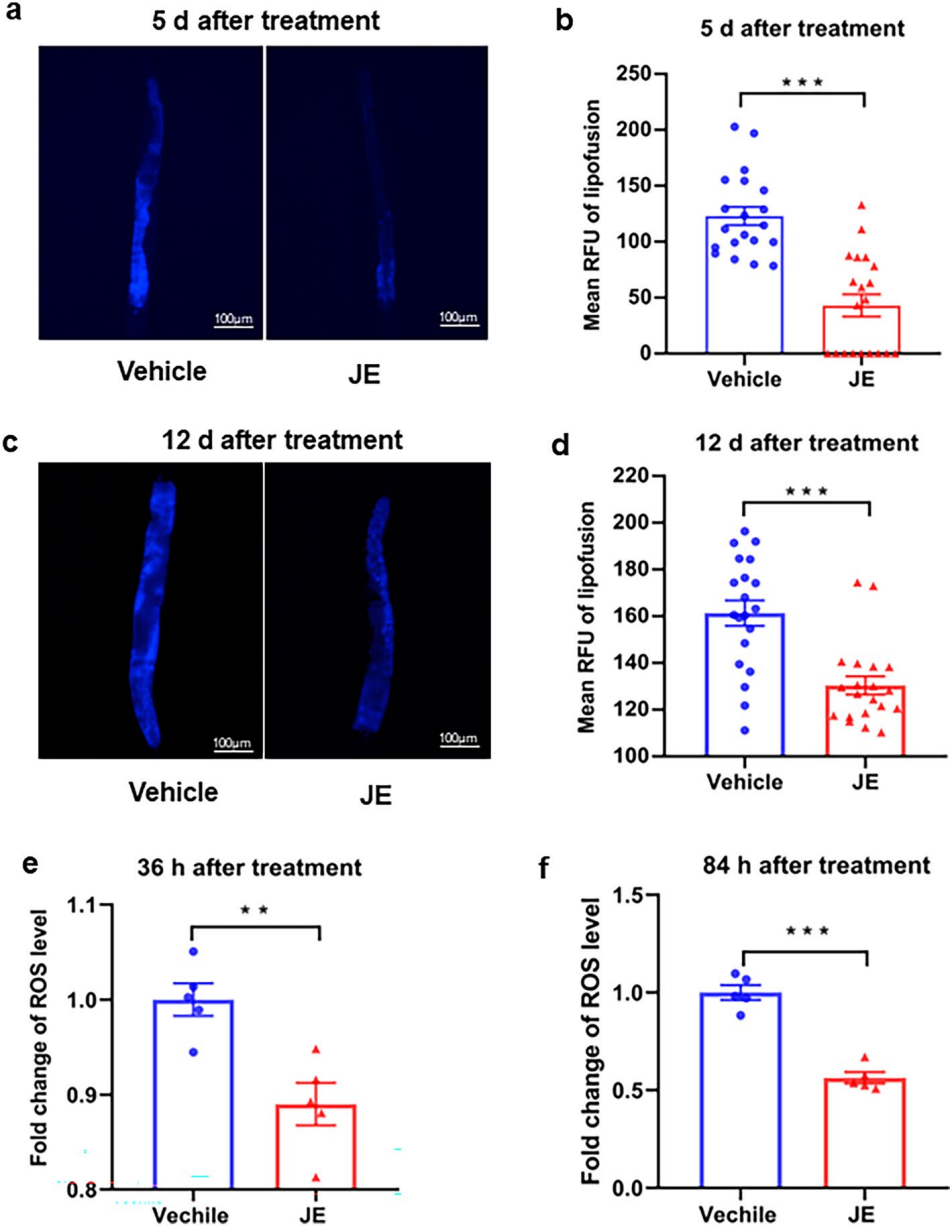
JE treatment decreases intestinal autofluorescence (lipofuscin) and ROS levels in adult wild-type worms. (**a**) day 5 adult wild-type worms. (**b**) Mean RFU value. (n = 20) (**c**) day 12 adult wild-type worms. (**d**) Mean RFU value. (n = 20) (**e**) Assessment of ROS level with 36 h JE or vehicle treatment. (n = 5) (**f**) Assessment of ROS level with 84 h JE or vehicle treatment. (n = 5). Values are mean ± SE. *P ≤ 0.05, **P ≤ 0.01 ***P ≤ 0.001.

As a marker of aging and oxidative damage, the rate of lipofuscin formation increases with age and it depends on the rate of oxidative damage^7,38,39^. We detected lipofuscin levels of 100 μg/mL JE-treated or vehicle-treated wild-type *C. elegans* at day 5 and day 12 of adulthood. The result showed that, after receiving 5 or 12 days of JE treatment, the lipofuscin level in the intestine decreased by 65% and 20% respectively (Fig. 3a–d). To investigate the effect of JE treatment on oxidative damage, intracellular ROS levels were evaluated in wild-type worms using H2DCF-DA, a widely known fluorescence probe for detecting intracellular ROS production. The results displayed that, with 2 or 4 days of JE treatment, the ROS accumulation of the wild-type worms had a significant decrease (Fig. 3e,f).

### Effect of JE on pharyngeal pumping rate and fertility of *C. elegans*

**Figure 4:**
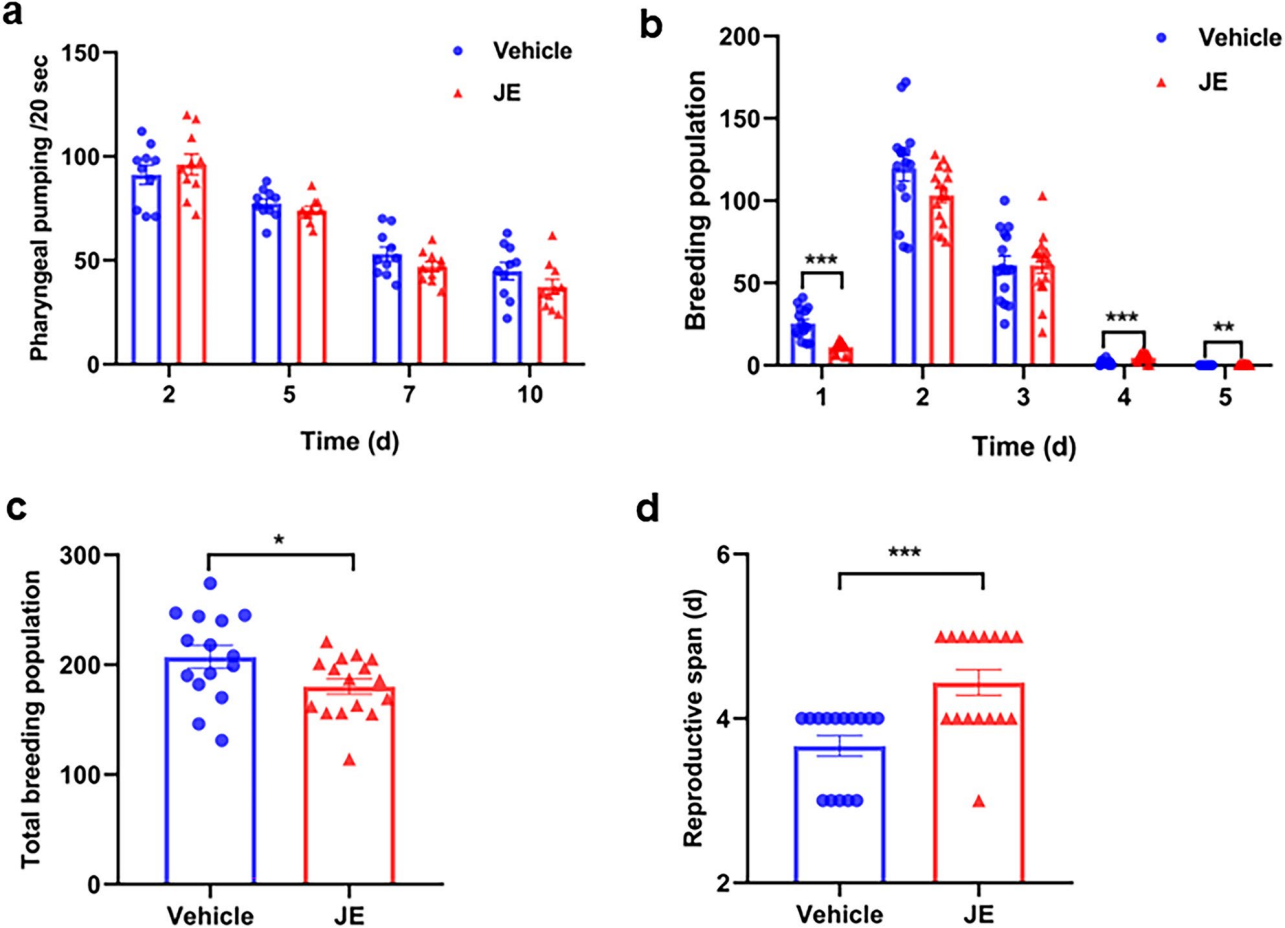
Effect of JE on the fertility of *C. elegans*. (**a**) Mean pumping rates (pumps per 20 s) are shown for each time point (n = 10). (**b**, **c**) the number of eggs laid of *C. elegans* treated with 100 μg/mL JE (n = 16) or vehicle (n = 15). (**d**) breeding period of *C. elegans* treated with 100 μg/mL JE (n = 16) or vehicle (n = 15). Values are mean ± SE. *P ≤ 0.05, **P ≤ 0.01 ***P ≤ 0.001.

Worms with reduced pharyngeal pumping ingest fewer bacteria and exhibit numerous DR-like characteristics, such as decreased fecundity and prolonged lifespan^5^. We tested whether JE had an effect on the pharyngeal pumping rate, and found that, compared to the control, 100 μg/mL JE treatment had no effect on pharyngeal pump rates. This indicated that JE treatment did not affect the feeding behavior of the worms. Next, we explored whether the extension in lifespan was accompanied by any effect on the fertility of nematodes. The total offspring per worm in the control group was 207.2 ± 10.33. After administering 100 µg/mL JE, the total offspring decreased to 180.19 ± 7 (Fig. 4b,c). To our surprise, the mean breeding days increased by 21% (3.67 days in control worms, 4.44 days in 100 ug/ml JE treatment worms, p < 0.001) (Fig. 4d). Therefore, these results manifested that, with the lifespan extension, the JE treatment significantly decreased fertility and increased the breeding period of worms.

### Effect of JE on the mobility of *C. elegans*

**Figure 5:**
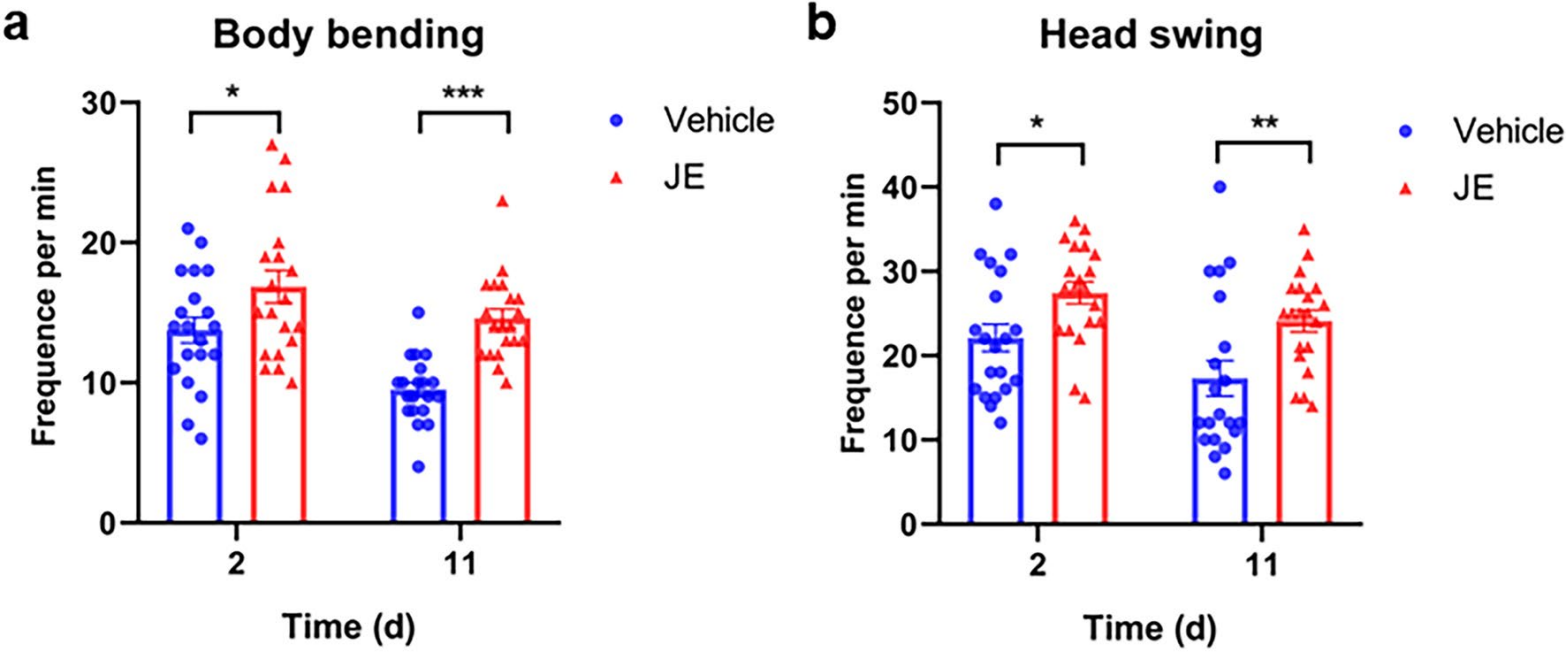
JE increases the motility of *C. elegans*. (**a**) The counts of body bending (n = 20). (**b**) The counts of head swing (n = 20). Values are mean ± SE. *P ≤ 0.05, ***P ≤ 0.001.

With the aging process, the worm's activity slows down and becomes insensitive to external stimuli^40,41^. Stamper and Hosono previously reported that wild-type worms exhibit an age-dependent decline in movement ability, the decline of activity was rapid from days 7–10^41,42^. To investigate whether the increased lifespan was accompanied by the improvement of movement behavior, we conducted a movement behavior assay (body bending and head swing) at the age of 2 and 11 days of L4 stage. The results showed that the head swing and body bending of the JE treated group were significantly higher than those of the control group on day 2 and day 11. With the increase of age, the body bending frequency of *C. elegans* decreased by 30.9% (from 13.75 to 9.5 times per minute), and that of *C. elegans* in JE group decreased by 13.35% (from 16.58 to 14.6 times per minute) (Fig. 5a). Similarly, head swing decreased from 22.1 to 17.3 times per minute in *C. elegans* and from 27.45 to 24.1 times per minute in JE group, with a decrease of 21.7% and 12.2%, respectively (Fig. 5b).

### JE requires *daf-16* to extend the lifespan of *C. elegans*

**Figure 6:**
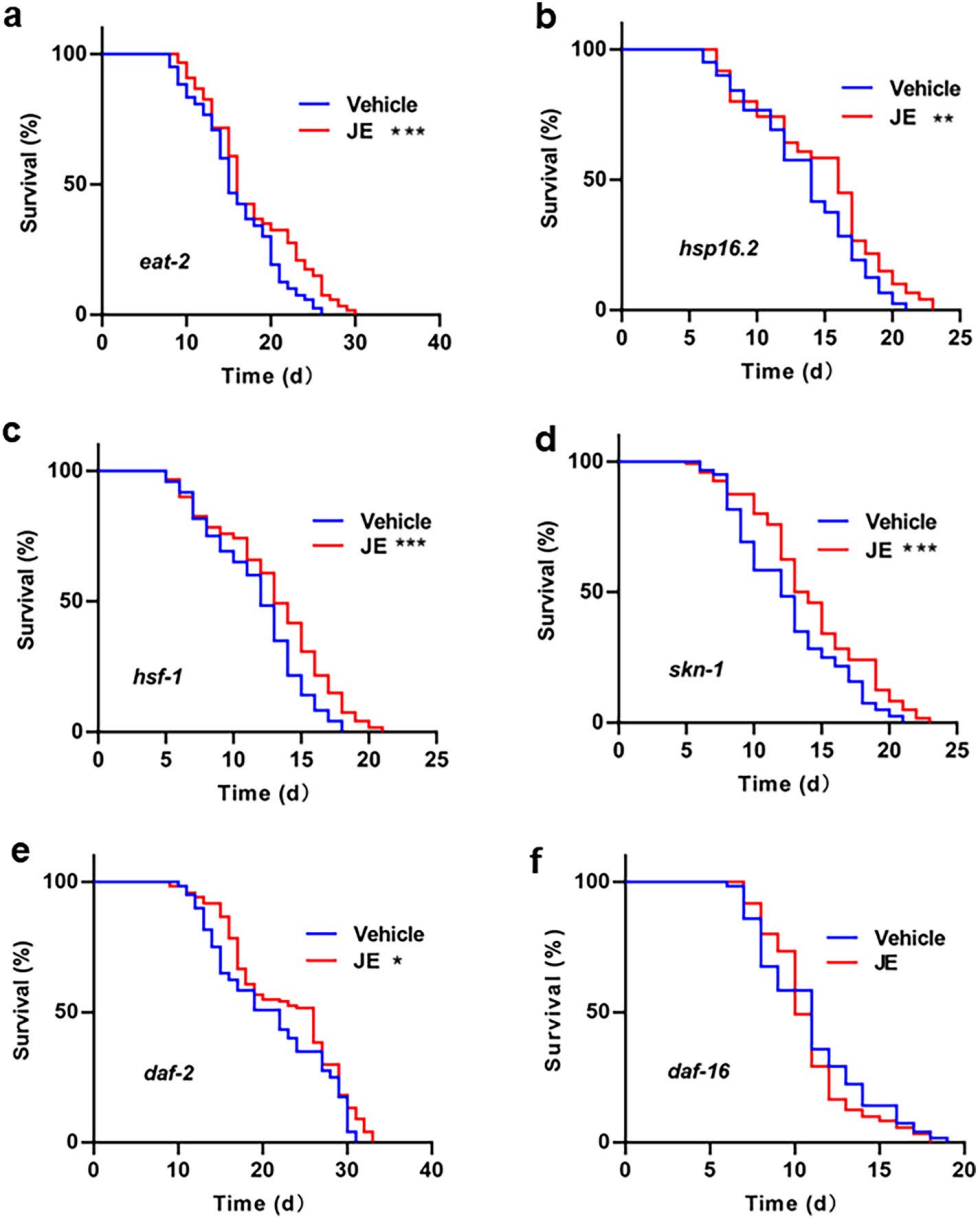
Effect of JE on Lifespan of mutant *C. elegans*. (**a**) The lifespan of *eat-2* mutant treated with 100 μg/mL JE or vehicle (n = 120). (**b**) The lifespan of *hsp-16.2* treated with 100 µg/mL JE or vehicle (n = 120). (**c**) The lifespan of *hsf-1*mutant treated with 100 μg/mL JE or vehicle (n = 120). (**d**) The lifespan of *skn-1*mutant treated with 100 μg/mL JE or vehicle (n = 120). (**e**) The lifespan of *daf-2* mutant treated with 100 µg/mL JE or vehicle (n = 120). (**f**) The lifespan of *daf-16* mutant treated with 100 μg/mL JE or vehicle(n = 120). *P ≤ 0.05, **P ≤ 0.01.

In order to investigate the molecular mechanisms of JE on longevity extension and health improvement in *C. elegans*, we next dissected the longevity pathways required for the lifespan extension induced by JE by testing its effects in prototypical mutant worms for aging-related signaling pathways such as insulin/insulin-like growth factors-1 (IIS), caloric restriction, ROS and so on. The results showed that both *eat-2* (caloric restriction)*, daf-2* (IIS)*, hsf-1*, *skn-1* and *hsp-16.2* mutant worms had an increased mean lifespan and maximum lifespan with JE treatment as compared with their respective mutant vehicle groups (Fig. 6a–e, Tables S4–S9). In contrast, the effect of JE on lifespan was dependent on insulin/IGF1 signal pathway, as the improvement was entirely suppressed in *daf-16* mutants, with the 100 ug/ml JE treatment, the mean lifespan and the maximum lifespan of *daf-16* mutant worms had no change (Fig. 6f). All together, these results indicated that JE treatment requires *daf-16* gene to extend the mean and maximum lifespan of *C. elegans*.

### JE cannot affect the mobility, fertility, and lifespan under stress resistance of mutant *C. elegans* (*daf-16*)

**Figure 7:**
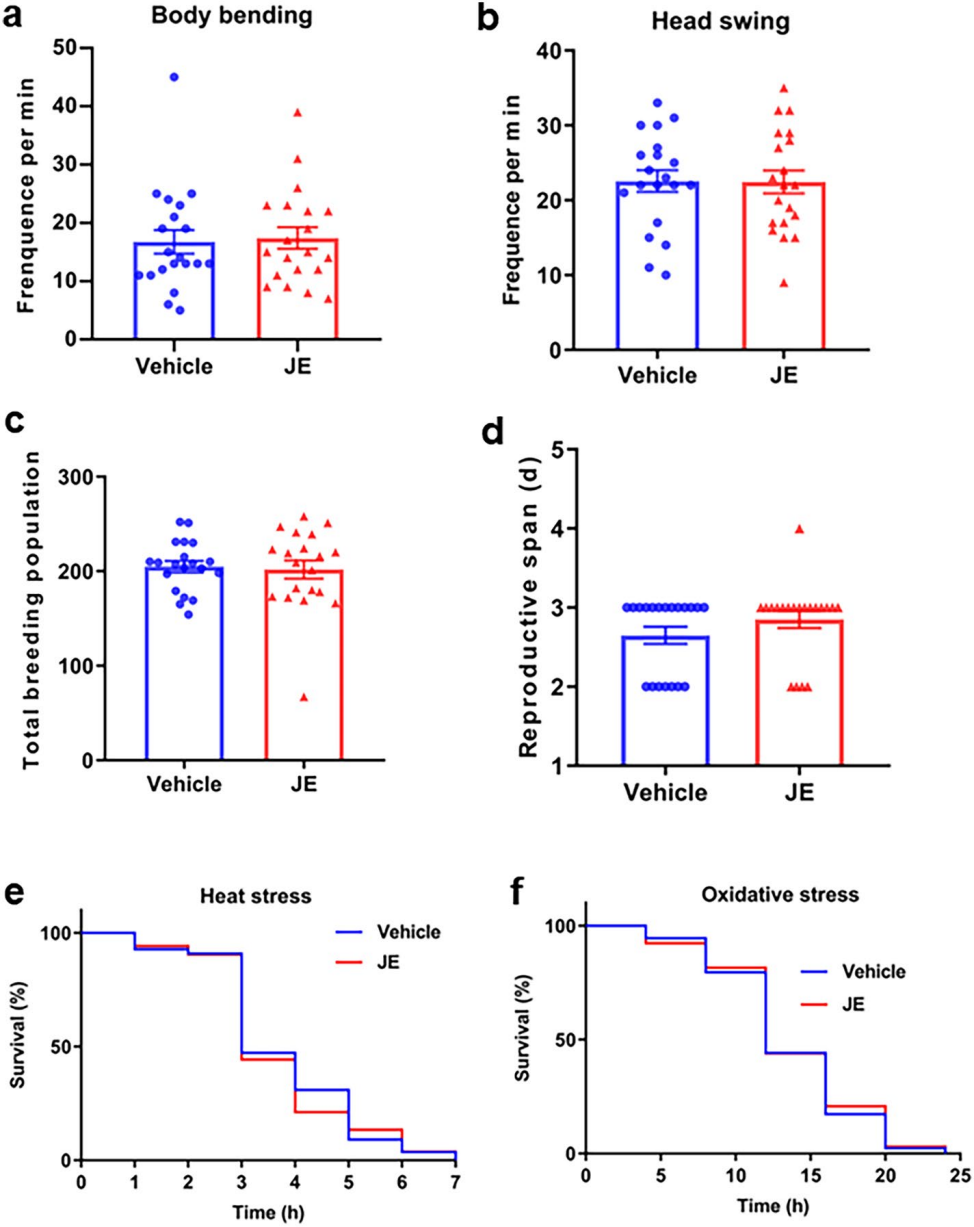
Effect of JE on the motility, fertility and resistance to stress of *daf-16* mutant *C. elegans*. (**a**) body bending (n = 20). (**b**) head swing (n = 20). (**c**) number of eggs laid (n = 20). (**d**) breeding period (n = 20). (**e**) Exposed to 37 °C thermal shock (n ≥ 52). (**f**) Exposed to 50 μM paraquat (n ≥ 127). Values are mean ± SE. *P ≤ 0.05.

Next, we observed the effect of JE on physiological indexes of *daf-16* mutant *C. elegans*. In the vehicle group, the worms bent their bodies 16.8 ± 2.0 times and swing heads 22.6 ± 1.5 times per minute, compared with 17.4 ± 1.9 and 22.5 ± 1.5 times per minute in the JE treatment group (Fig. 7a,b). Similarly, JE treatment cannot significantly affect the number of eggs laid and breeding period of *daf-16* mutant *C. elegans*. Under the treatment of JE, *daf-16* mutant *C. elegans* breed 258 ± 19.7 eggs total, and the oviposition duration was 3.1 ± 0.2 days, which were not significantly different from the control group (Fig. 7c,d). Then, the mutant worms were treated with JE for 48 h and placed at 37 °C and paraquat to observe their resistance to heat stress and oxidative stress. Our results showed that JE did not significantly increase the mean lifespan and maximum lifespan of *daf-16* mutants under heat and oxidative stress conditions (Fig. 7e,f, Tables S10–S11).

### Effect of JE treatment on DAF-16 translocation

**Figure 8:**
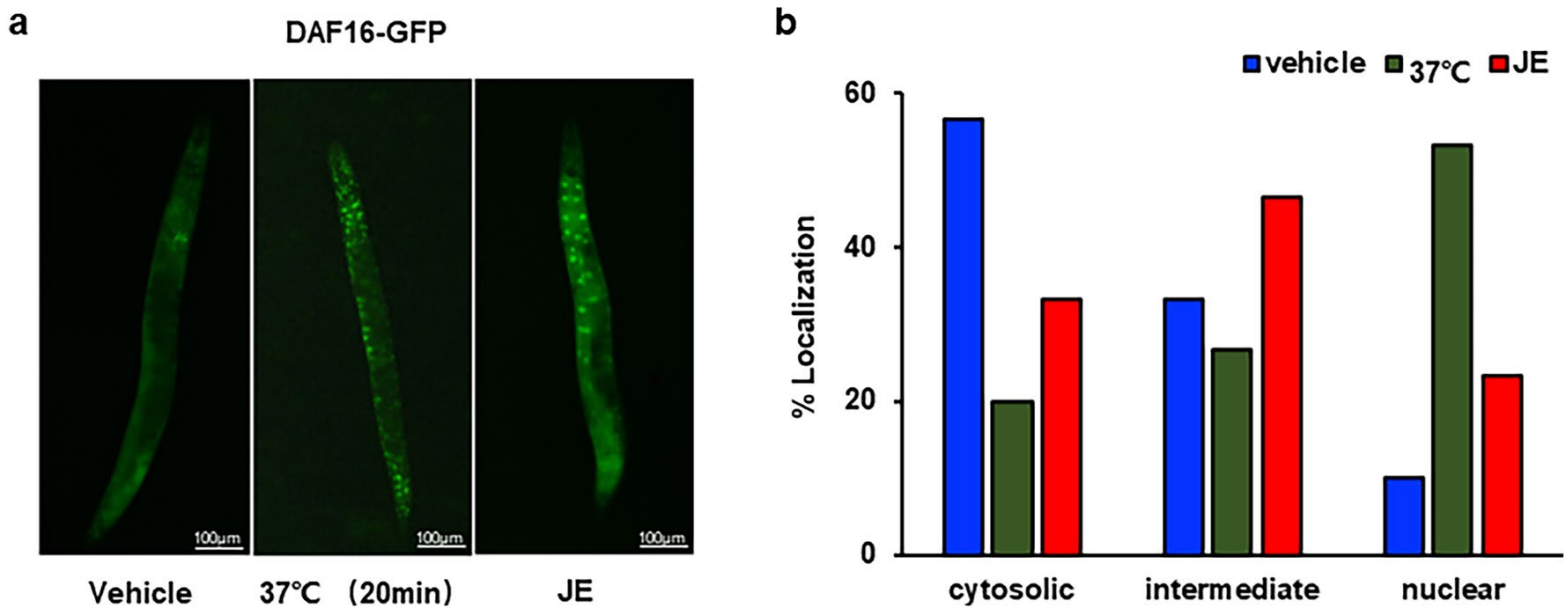
Effect of JE on daf-16 of *C. elegans*. (**a**) Representative images of GFP in TJ356 (DAF16-GFP) worm treated with vehicle, heat shock (37 °C, 20 min) and 100 μg/mL JE. (**b**) DAF-16 translocation assay (n = 30).

The JE treatment did not change the lifespan, mobility, and fertility of *daf-16* mutant worms, it indicated that the effects of JE treatment depend on the gene *daf-16* in *C. elegans*. DAF-16 is a key factor in insulin/IGF1 signaling pathways transferred from the cytoplasm to the nucleus for multiple biological processes under stress. To determine whether JE is able to affect the cellular localization of DAF-16, we introduced the green fluorescent protein GFP-tagged *daf-16* transgenic strain TJ356 to observe the location of DAF-16. The result showed that DAF-16::GFP of 9% transgenic worms localized in the nucleus with vehicle treatment, but heat stress and JE treatment translocates DAF-16::GFP to nucleus in transgenic worms (Fig. 8a,b).

### JE requires *sod-3 *(the downstream of *daf-16*) to extend the lifespan of *C. elegans*

**Figure 9:**
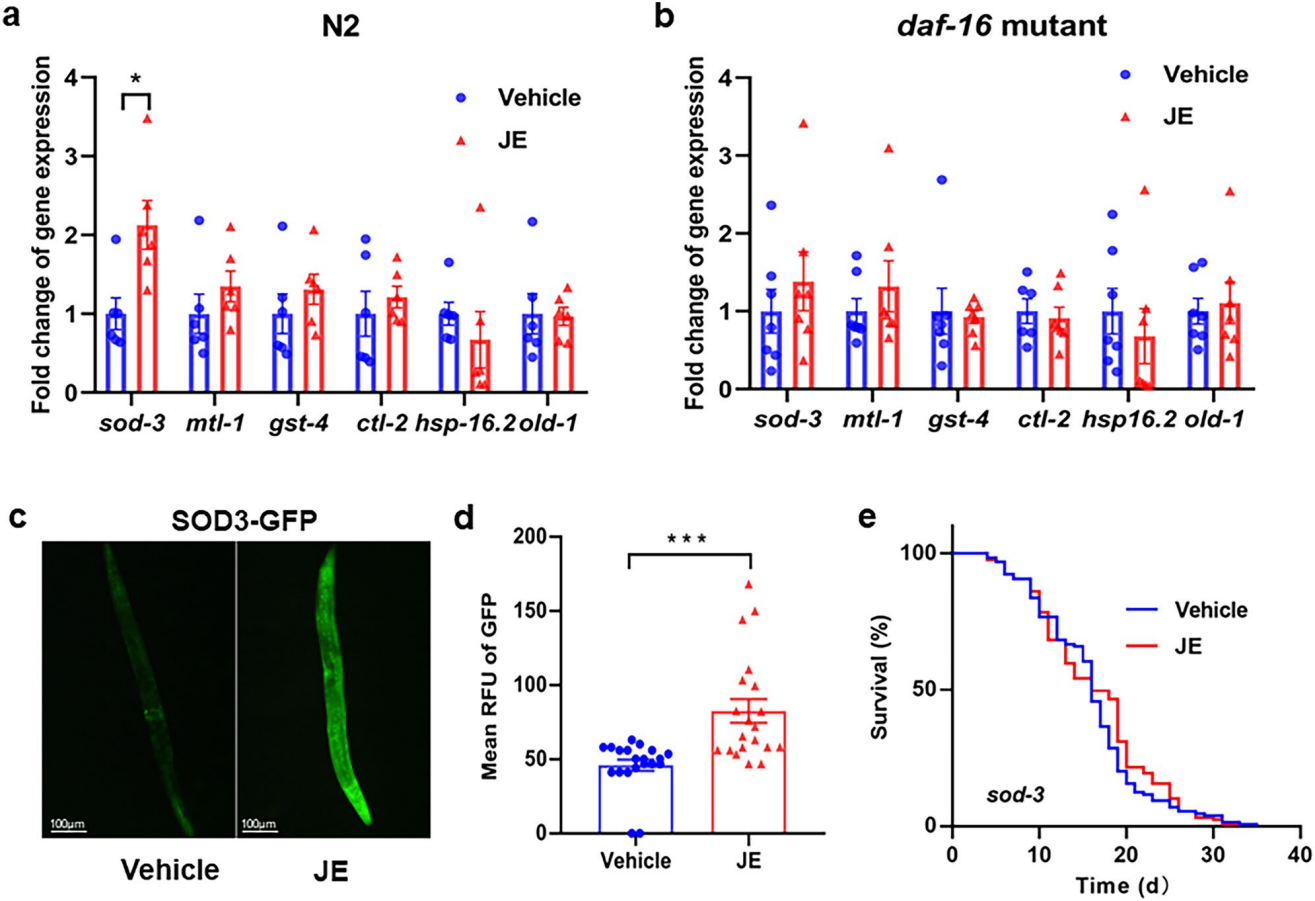
Genes affected by JE treatment in *C. elegans* wild-type. (**a**) The relative expression level of the downstream gene of *daf-16* in N2 strain after JE treatment. (n = 6). (**b**) The relative expression level of the downstream gene of *daf-16* in GR1307 strain (*daf-16* mutant) after JE treatment. (n = 7). (**c**) Representative images of GFP in CF1553 (SOD3-GFP) worm treated with vehicle and 100 μg/mL JE. (**d**) The expression of SOD-3 assay (n = 20). (**e**) The lifespan of *sod-3* mutant treated with 100 μg/mL JE or vehicle (n = 129). Values are mean ± SE. **P ≤ 0.01, ***P ≤ 0.001.

To further explore the mechanism of *daf-16* with JE treatment, the expression levels of genes downstream of *daf-16* (*sod-3*, *mtl-1*, *gst-4*, *hsp-16.2*, *ctl-2*, *old-1*) were assessed. The expression level of genes downstream of *daf-16* in the N2 worms without JE treatment was set to 1. JE treatment increased the relative expression of *sod-3* by 2.1-fold, but had no influence on the expression of other *daf-16* downstream genes. (Fig. 9a). Meanwhile, we compared gene expression in *daf-16* mutant worms with the vehicle and JE treatment. Compared with vehicle group, the JE-treated groups did not show significant differences in the expression of *sod-3* (Fig. 9b). Furthermore, the GFP-tagged *sod-3* transgenic strain CF1553 (muls84) was introduced to investigate whether JE increased the protein level of *sod-3*. Our results showed that 100 μg/mL JE treatment significantly induced the expression of SOD-3 (Fig. 9c,d). In the present study, we found that JE treatment did not change the mean and maxium lifespan of *sod-3* mutant worms (Fig. 9e, Table S12). In conclusion, our results indicated that JE treatment was able to induce high expression of gene *sod-3* that might be depending on nuclear translocation of longevity-associated transcription factor DAF-16.

## Discussion

As a traditional Chinese medicine and food, *Jujubae Fructus* has been used in the treatment and late intervention of many diseases. However, there are few studies on its effect on aging. Here, we treated *C. elegans* with JE to observe the effects on longevity and health status, and further investigate the mechanisms involved. Our data showed that 50, 100 and 200 μg/mL of JE could significantly increase the lifespan of worms by 8.42%, 19.78% and 10.18%, respectively (Fig. 2b). Increased longevity has been accompanied by improved health status. We also observed how JE affected the lifetime of worms under stress, and the findings revealed that worms treated with JE had greater resilience to heat stress and oxidative stress (Fig. 2c,d). Many studies on *Jujubae Fructus* revealed various beneficial nutrients, including carbohydrate, mineral, vitamin, sugar and amino acid^43^, as well as various kinds of secondary metabolites, such as nucleotides (71.98 mg/100 g), flavonoids (48.62 mg/100 g), triterpenic acids (343.43 mg/100 g), polysaccharides (3.30 g/100 g)^44^. In this study, we identified 22 ingredients in the water extract of *Jujubae Fructus* by UPLC-MS analysis (Table 1). Among them, rutin treatment was reported to reduce polyglutamine (polyQ) protein aggregation in muscle and polyQ-mediated neuronal death in ASH sensory neurons, and extend lifespan in *C. elegans*. The possible mechanisms involved are antioxidant activity, activation of protein degradation (autophagy) and insulin/IGF1 signaling pathways^45^. As a representative triterpenic acid, ursolic acid prolonged the life span of *C. elegans*, and significantly lowered reactive oxygen species (ROS), also could act through serotonin receptors to enhance stress resistance^46^. Additionally, despite not identified, it is presumed that there were abundant polysaccharides in the JE prepared by decocting method, which were reported to be immunomodulating, antioxidative and hepatoprotective^4,47^. These bioactive constituents may contribute to the beneficial effects of prolonging lifespan and improving stress tolerance in *C. elegans* by JE in this study.

With the increase of age and oxidative stress, the level of lipofuscin in nematodes rises. Assessments of lipofuscin levels revealed that, in comparison to the control group, the JE group exhibited significantly lower levels of lipofuscin in nematodes on days 5 and 12 post-treatment. Subsequent assessments of ROS levels showed that, similarly, JE significantly reduced the levels of ROS in nematodes (Fig. 3). This suggests that JE can reduce the accumulation of lipofuscin and the levels of oxidative stress in nematodes during the aging process. Gagnon et al. found that telomere length in women who had given birth was significantly shorter than that of non-fertile women by analyzing the effects of fertility on the health status, and they indicated that reproduction may have accelerated the aging process^48^. Some studies have shown that extension of lifespan is correlated with a decrease in fecundity^49^. In addition, decreased worm fertility was associated with increased lifespan^24^. Removal of reproductive precursor cells in normal and *daf-2* mutant worms prolongs the lifespan of *C. elegans*^50,51^. Blueberry extract treatment with extended lifespan of nematodes can simultaneously reduce the number of eggs laid and prolong the reeding period of *C. elegans*^24^. Our results also showed that the *C. elegans* breeding period was prolonged, and the fecundity was decreased with the 100 ug/ml JE treatment (Fig. 4). Among long-lived humans, it was found that they had their last child at an older age, and the later the last child was born, the longer the females lived^52,53^. In our hand, with the extension of lifespan, the 100 µg/ml JE treatment increased by 0.77 days in breeding period of *C. elegans* (Fig. 4d). The aforementioned data indicate that JE can reduce oxidative damage in nematodes, enhance their mobility during the aging process, decrease fertility, and prolong reproductive preiod. These effects contribute to the beneficial impact of JE on delaying *C. elegans* aging.

Dietary restriction is one of the ways to delay the senescence of nematodes. *Eat-2* is a key gene in dietary restriction, it is a gene regulating pharyngeal suction rate, and its mutation will lead to feeding defects and reduce energy and nutrient intake of nematodes^54^. In order to observe whether the longevity of *C. elegans* was prolonged by dietary restriction, we examined the effect of JE on the longevity of *eat-2* mutants. The results showed that compared with the control group, JE significantly prolonged the mean and maximum lifespan of *eat-2* mutants (Fig. 6a, Table S4). Consistent with this, the result of pharyngeal pumping rate assay showed that JE treatment had no effect on pharyngeal pump rates on days 2 and 5 (Fig. 4a). Our results suggested that the dietary restriction pathway is nonessential for the effect of JE on the longevity of worms. There are other signaling pathways involved in the aging process of nematodes^55^. DAF-16 is homology with FoxO in mammals, and it is a key protein in nematode longevity^56^. Herein, to further study the molecule mechanism of prolongated lifespan and improved health status of *C. elegans* with the JE supplementation, we investigated whether JE affected the longevity of nematodes deficient in *daf-2*, *hsf-1*, *skn-1*, *hsp-16.2* and *daf-16*, respectively. However, compared with the control group, the life-prolonging effect of JE only disappeared in the mutant nematode *daf-16*. This suggests that the presence of DAF-16 is necessary for JE to prolong the lifespan of nematodes. Next, we observe the effects of JE on vitality, fertility, and stress resistance in mutant nematode *daf-16*. Consistently, the effects of JE on these indices in wild-type nematodes were not found in *daf-16* mutant nematodes (Fig. 7a–f, Tables S5–S9). These findings suggested that the activity of *daf-16* gene is crucial for JE to prolong the lifespan and improve the health status of worms. Normally, DAF-16 is located in the cytoplasm, while under stress, DAF-16 is transferred from the cytoplasm to the nucleus, JE treatment also translocate DAF-16::GFP to nucleus in TJ356 worms compared to those untreated ones (Fig. 8a,b). Under normal physiological activity, the activity of DAF-16 is at a relatively low level. In a stress environment, the IIS signaling pathway receives signals that reduce its activity, DAF-16 migrates from the cytoplasm to the nucleus, activating a large number of stress resistant and longevity gene expressions, which prolongs the lifespan of *C. elegans*^57^.

To investigate whether the enhancement of nuclear localization of DAF-16 by JE activates the expression of downstream resistance genes, thereby positively affecting the lifespan of nematodes, we examined the expression of downstream genes of *daf-16*. The results showed that JE treatment increased the expression of *sod-3*, downstream of *daf-16* in N2 worms, but the effect was inhibited in mutant (*daf-16*) *C. elegans*. We also found that JE treatment doesn’t significantly increase the lifespan and the expression of *sod-3* of mutant (*daf-16*) worms as in wild type (Fig. 9a,b). Our further study manifested that JE’s effect on prolonging the lifespan of worms disappeared with the silencing gene of *sod-3* (Fig. 9e)*. Sod-3* encodes a superoxide dismutase that can resist oxidative stress and prolong life. Trilobatin has been shown to effectively extend the lifespan of nematode worms by regulating the SKN1/SIRT3/DAF16 signaling pathway, as well as increasing the activity of antioxidant enzymes CAT and SOD-3^58^. Curcumin acetylsalicylate can also delay the aging of nematodes by activating the expression of *daf-16* and its downstream antioxidant genes *sod-3* and *gst-4*^59^. Our findings indicated that JE treatment may induce nuclear translocation of longevity associated transcription factor DAF-16 depending on activation of downstream gene *sod-3* to prolong lifespan of worms. Meanwhile, the specific small molecule in JE that prolongs the lifespan of *C. elegans* and its mechanism, as well as the important role of *daf-16* in those effects, need further investigation. *Jujubae Fructus* has been poorly studied in terms of aging, and previous studies have reported a life-extending effect in drosophila^16^. Our study makes up for the gap in the impact of *Jujubae Fructus* on lifespan of *C. elegans* and provides a certain basis for the relative studies on humans.

## Conclusion

This study, for the first time, reports *Jujubae Fructus* extract supplementation promotes lifespan and improves the health status of the *C. elegans* model system. We identified 22 phytochemicals in JE, including three carbohydrates, five glycosides, two alkaloids, eleven triterpene acids and cAMP. Our data showed that JE treatment has a beneficial effect on delaying aging in *C. elegans*. JE can prolong the lifespan of nematodes under standard laboratory environmental conditions, enhance their vitality, and extend their reproductive period. In addition, JE also enhances the resistance of nematodes to stressful environments. Based on specific mutation studies, the beneficial effects of JE on aging depend on DAF-16. JE may exert beneficial effects by promoting the transfer of DAF-16 from the cytoplasm to the nucleus and thereby promoting the expression of downstream stress resistance genes. As a downstream gene of *daf-16*, *sod-3* is critical to the beneficial effects of JE. Overall, JE enhances the stress resistance of nematodes under stress environments by promoting the nuclear translocation of DAF-16 to promote the expression of the stress resistance gene *sod-3*, and delays the aging of nematodes under normal and stress conditions. Our study makes up for the gap in the impact of *Jujubae Fructus* on lifespan of *C. elegans*, the present study may provide a novel avenue against aging and aging related disorders.

### Supplementary Information

Supplementary Information.

Supplementary Tables.

## Data Availability

All data generated or analysed during this study are included in this published article [and its supplementary information files].

## Abbreviations

FUDR: Fluoro-2ʹ-deoxy-β-uridine
JE: Jujubae Fructus extract
H2DCF-DA: 2′,7′-Dichlorodihydroflfluoresceindiacetate
IDA: Information-dependent acquisition
NGM: Nematode growth medium
PDAD: Photodiode array data
ROS: Reactive oxygen species
TCMSP: Traditional Chinese medicine systems pharmacology
TIC: Total ions chromatogram
