# Supplementary material for: Jujubae Fructus extract prolongs lifespan and improves stress tolerance in Caenorhabditis elegans dependent on DAF-16/SOD-3
Source: Sci Rep. 2024 Jun 14;14:13713. doi: 10.1038/s41598-024-64045-0 (PMC11178930; doi:10.1038/s41598-024-64045-0)
Supplement: Supplementary file 1 — Supplementary Information. [file 41598_2024_64045_MOESM1_ESM.docx]

Supplementary Material


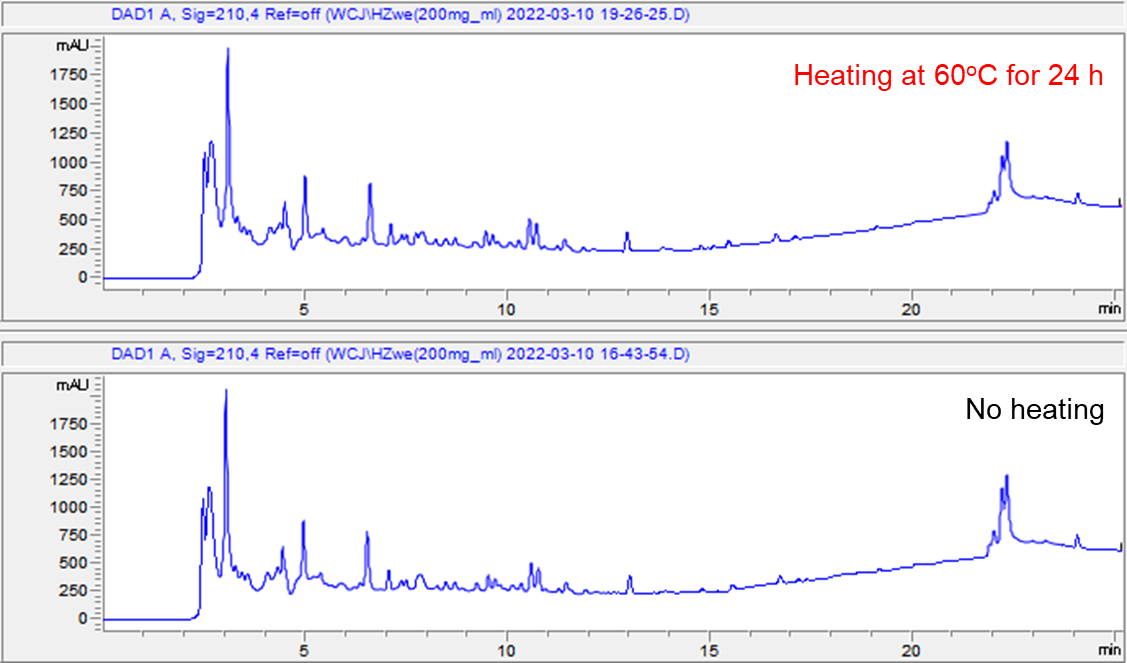


**Figure S1.** The chemical stability test for JE aqueous solution by HPLC-PDAD-UV analysis


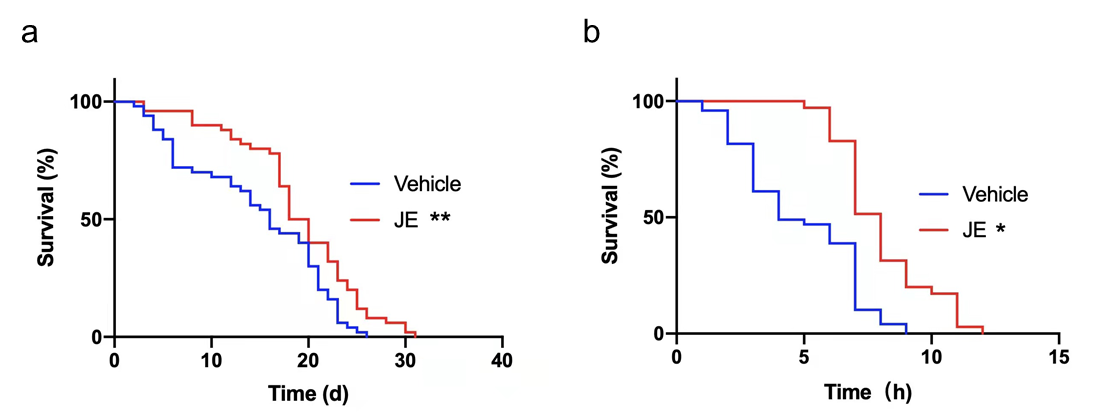


**Figure S2.** Effect of JE on *C. elegans* feeding dead OP50. Lifespan of *C. elegans* treated with 100 μg/mL JE or vehicle, (a) at normal condition (20℃), (b) exposed to 37°C thermal shock. (n = 50). Values are mean ± SE. *P ≤ 0.05, **P ≤ 0.01.

| **Table S1.** Effect of JE on the lifespan of N_2_ *C. elegans* | | | | | |
| --- | --- | --- | --- | --- | --- |
| Group | Number | Mean lifespan (days) | % of control | P value VS Control | Maximum lifespan (days) |
| Vehicle | 146 | 15.76±3.59 | 100 | —— | 21 |
| JE (20μg/ml) | 149 | 16.18±3.67 | 102.6 | 0.3602 | 21 |
| JE (50μg/ml) | 148 | 17.25±4.77 | 109.4 | 0.0029 | 24 |
| JE (100μg/ml) | 150 | 19.12±5.42 | 121.3 | <0.001 | 27 |
| JE (200μg/ml) | 150 | 17.60±5.30 | 111.6 | <0.001 | 27 |

| **Table S2.** JE extend the lifespan of N_2_ under heat stress | | | | | |
| --- | --- | --- | --- | --- | --- |
| Group | Number | Mean lifespan (hours) | % of control | P value VS Control | Maximum lifespan (hours) |
| Vehicle | 121 | 7.58±2.90 | 100 | —— | 13 |
| JE (100μg/ml) | 120 | 8.81±5.30 | 116.13 | 0.0014 | 17 |

| **Table S3.** JE extend the lifespan of N_2_ under oxidative stress | | | | | |
| --- | --- | --- | --- | --- | --- |
| Group | Number | Mean lifespan (hours) | % of control | P value VS Control | Maximum lifespan (hours) |
| Vehicle | 160 | 12.48±2.94 | 100 | —— | 22 |
| JE (100μg/ml) | 139 | 8.81±5.30 | 107.00 | 0.021 | 22 |

| **Table S4.** JE extend the lifespan of *eat-2* mutant *C. elegans* | | | | | |
| --- | --- | --- | --- | --- | --- |
| Group | Number | Mean lifespan (days) | % of control | P value VS Control | Maximum lifespan (days) |
| Vehicle | 119 | 15.99±4.82 | 100 | —— | 26 |
| JE (100μg/ml) | 120 | 17.82±5.72 | 111.41 | 0.0080 | 30 |

| **Table S5.** JE extend the lifespan of *hsf-1* mutant *C. elegans* | | | | | |
| --- | --- | --- | --- | --- | --- |
| Group | Number | Mean lifespan (days) | % of control | P value VS Control | Maximum lifespan (days) |
| Vehicle | 120 | 11.70±3.56 | 100 | —— | 18 |
| JE (100μg/ml) | 120 | 12.96±4.24 | 110.75 | 0.0145 | 21 |

| **Table S6.** JE extend the lifespan of *hsp16.2* mutant *C. elegans* | | | | | |
| --- | --- | --- | --- | --- | --- |
| Group | Number | Mean lifespan (days) | % of control | P value VS Control | Maximum lifespan (days) |
| Vehicle | 120 | 13.55±4.15 | 100 | —— | 21 |
| JE (100μg/ml) | 120 | 14.71±4.73 | 108.55 | 0.0452 | 23 |

| **Table S7.** JE extend the lifespan of *skn-1* mutant *C. elegans* | | | | | |
| --- | --- | --- | --- | --- | --- |
| Group | Number | Mean lifespan (days) | % of control | P value VS Control | Maximum lifespan (days) |
| Vehicle | 120 | 12.48±3.97 | 100 | —— | 21 |
| JE (100μg/ml) | 120 | 14.15±4.34 | 113.35 | 0.0018 | 33 |

| **Table S8.** JE extend the lifespan of *daf-2* mutant *C. elegans* | | | | | |
| --- | --- | --- | --- | --- | --- |
| Group | Number | Mean lifespan (days) | % of control | P value VS Control | Maximum lifespan (days) |
| Vehicle | 119 | 20.92±6.90 | 100 | —— | 31 |
| JE (100μg/ml) | 120 | 22.83±6.75 | 109.13 | 0.0313 | 33 |

| **Table S9.** JE extend the lifespan of *daf-16* mutant *C. elegans* | | | | | |
| --- | --- | --- | --- | --- | --- |
| Group | Number | Mean lifespan (days) | % of control | P value VS Control | Maximum lifespan (days) |
| Vehicle | 120 | 10.98±3.28 | 100 | —— | 19 |
| JE (100μg/ml) | 120 | 10.8±2.62 | 98.41 | 0.6613 | 18 |

**Table S10.**The information of the primer sequence for q-PCR

| Gene | primer sequence（5’- 3’） |
| --- | --- |
| *β-actin*-F | GCTCTTGCCCCATCAACCAT |
| *β-actin*-R | GCCGGACTCGTCGTATTCTT |
| *sod-3*-F | TCTACTGCTCGCACTGCTTC |
| *sod*-3-R | CTGGGAGAGTGTGCTTGGAG |
| *ctl-2-*F | AGTTTGGCCACACGGTGATT |
| *ctl-2*-R | AAGGCGGTGGAAATGAGTGT |
| *gst-4*-F | AAGCTGAAGCCAACGACTCC |
| *gst-4*-R | AATGGGAAGCTGGCCAAATG |
| *mtl-1*-F | GGCTTGCAAGTGTGACTGC |
| *mtl-1*-R | TCTCCGCACTTGCATTGCTT |
| *hsp-16.2*-F | TCCATCTGAGTCTTCTGAGATTGTT |
| *hsp-16.2*-R | TGAGACGTTGAGATTGATGGCA |
| *old-1*-F | TGCTGCTGATTTTCTTTCCATT |
| *old-1*-R | TGAGGAAGAGGAATCAAGTGAGG |
