# Supplementary material for: Jujubae Fructus extract prolongs lifespan and improves stress tolerance in Caenorhabditis elegans dependent on DAF-16/SOD-3
Source: Sci Rep. 2024 Jun 14;14:13713. doi: 10.1038/s41598-024-64045-0 (PMC11178930; doi:10.1038/s41598-024-64045-0)
Supplement: Supplementary file 2 — Supplementary Tables. [file 41598_2024_64045_MOESM2_ESM.docx]

| **Table S10.** JE extend the lifespan of *daf-16* mutant under heat stress | | | | | |
| --- | --- | --- | --- | --- | --- |
| Group | Number | Mean lifespan (± SD) (hours) | % of control | P value VS Control | Maximum lifespan (hours) |
| Vehicle | 55 | 3.75±1.38 | 100 | —— | 7 |
| JE (100μg/ml) | 52 | 3.67±1.37 | 98.07 | 0.7764 | 7 |

| **Table S11.** JE extend the lifespan of *daf-16* mutant under oxidative stress | | | | | |
| --- | --- | --- | --- | --- | --- |
| Group | Number | Mean lifespan (± SD) (hours) | % of control | P value VS Control | Maximum lifespan (hours) |
| Vehicle | 127 | 17.92±2.44 | 100 | —— | 24 |
| JE (100μg/ml) | 130 | 18.25±2.53 | 101.85 | 0.6409 | 24 |

| **Table S12.** JE extend the lifespan of *sod-3* mutant *C. elegans* | | | | | |
| --- | --- | --- | --- | --- | --- |
| Group | Number | Mean lifespan (± SD) (days) | % of control | P value VS Control | Maximum lifespan (days) |
| Vehicle | 129 | 16.71±5.64 | 100 | —— | 35 |
| JE (100μg/ml) | 129 | 17.242±6.17 | 103.19 | 0.2479 | 35 |

**Table S13.**The information of the primer sequence for q-PCR

| Gene | primer sequence（5’- 3’） |
| --- | --- |
| *β-actin*-F | GCTCTTGCCCCATCAACCAT |
| *β-actin*-R | GCCGGACTCGTCGTATTCTT |
| *sod-3*-F | TCTACTGCTCGCACTGCTTC |
| *sod*-3-R | CTGGGAGAGTGTGCTTGGAG |
| *ctl-2-*F | AGTTTGGCCACACGGTGATT |
| *ctl-2*-R | AAGGCGGTGGAAATGAGTGT |
| *gst-4*-F | AAGCTGAAGCCAACGACTCC |
| *gst-4*-R | AATGGGAAGCTGGCCAAATG |
| *mtl-1*-F | GGCTTGCAAGTGTGACTGC |
| *mtl-1*-R | TCTCCGCACTTGCATTGCTT |
| *hsp-16.2*-F | TCCATCTGAGTCTTCTGAGATTGTT |
| *hsp-16.2*-R | TGAGACGTTGAGATTGATGGCA |
| *old-1*-F | TGCTGCTGATTTTCTTTCCATT |
| *old-1*-R | TGAGGAAGAGGAATCAAGTGAGG |
